# Low Density Lipoprotein - important player in increasing cryoprotective efficiency of soybean lecithin-based bull semen extenders

**DOI:** 10.21451/1984-3143-AR2018-0107

**Published:** 2019-10-23

**Authors:** Šimoník Ondřej, Šichtař Jiří, Beran Jan, Maňásková-Postlerová Pavla, Tůmová Lucie, Martina Doležalová, Folková Petra, Stádník Luděk, Rajmon Radko

**Affiliations:** 1 Department of Veterinary Sciences, Faculty of Agrobiology, Food and Natural Resources, Czech University of Life Sciences Prague, Prague, Czech Republic.; 2 Department of Animal Husbandry, Faculty of Agrobiology, Food and Natural Resources, Czech University of Life Sciences Prague, Prague, Czech Republic.; 3 Institute of Biotechnology of the Czech Academy of Sciences, Laboratory of Reproductive Biology, v.v.i., Biocev, Průmyslová, Czech Republic.; 4 Department of Zootechnical Sciences, Faculty of Agriculture, University of South Bohemia in České Budějovice, České Budějovice, Czech Republic.

**Keywords:** cryopreservation, Low Density Lipoprotein, spermatozoa

## Abstract

Currently, considering cryopreservation of bull semen, there is no clear consensus over the comparability of cryoprotective efficacy of extenders with soybean lecithin and those based on egg yolk. The objective of this study was to prove the use of Low Density Lipoprotein (LDL) extracted from hen-egg yolk as an enhancing factor for soybean lecithin-based extenders. In total, 35 ejaculates of (seven bulls x five ejaculates per bull) were collected and cryopreserved at a commercial insemination centre. The effect of the LDL addition to the extenders AndroMed^®^ and Bioxcell^®^ was tested in a 6% (v/v) concentration on spermatozoa after thawing. Modified extender composition effects were assessed on sperm functional parameters motility, plasma membrane, mitochondrial membrane potential and acrosomal integrity after thawing by CASA, flow cytometry and fluorescent microscopy, respectively. Based on kinematic parameters determined from CASA, *k*-means cluster analysis was used to classify individual spermatozoon into specific subpopulations (fast, medium fast and slow). A subpopulation of fast spermatozoa was increased in the presence of LDL in both selected extenders (P < 0.05). Moreover, the positive effect of LDL on sperm motility was confirmed by decreasing the percentage of sperm in slow subpopulation (P < 0.05). The effect of LDL addition on the incidence of spermatozoa with intact plasma membrane was not demonstrated in any case of extender used (P > 0.05). The percentage of sperm with intact acrosome was improved when LDL was added to Bioxcell^®^ extender (P < 0.05). On the other hand, addition of LDL to AndroMed^®^ extender improved mitochondrial intactness after thawing (P < 0.05). In conclusion, our results showed that adding LDL to selected soybean lecithin-based extenders considerably ameliorated the functional parameters of spermatozoa after thawing and thus this lipoprotein could represent an improving agent for soybean lecithin-based extender for bull semen cryopreservation.

## Introduction

Artificial insemination (AI) in dairy cattle is primarily done with cryopreserved semen. Currently, it is generally accepted that cryopreservation of bull semen is adequately successful, however current protocols provide just a 50% recovery rate post-thawed (Layek et *al*., 2016). Thus, further improvement in cryopreservation protocol will provide economic advantages for the breeding industry. The cryopreservation process exposes spermatozoa to un-physiological conditions with adverse effects on their function. The plasma membrane (PM) structure is primarily damaged by harsh conditions during cryopreservation (Tapia *et al*., 2012). Moreover, PM status is, in close relationship to other sperm structures, essential for their proper physiological functions such as acrosome and mitochondria (Breitbart *et al*., 2005; Amaral *et al*., 2013). One of the main changes during cryopreservation is cholesterol losses from PM (Srivastava *et al*., 2013) which induces changes in its liquid state (Layek et al., 2016). Thus cholesterol obviously plays substantial role in the physiological functions of PM (Horokhovatskyi *et al*., 2016). Its increased content positively affects PM resistance to cryopreservation and, in other words, causes lower sensitivity of sperm to cryopreservation (Srivastava *et al*., 2013). One of the main method for reducing the negative impact of cryopreservation and to increase quality of insemination doses (ID) is an adjustment or modification of semen extender composition (Yeste, 2016). Generally, one of basic components of the extenders is those high-molecular weight non-permeable compounds mainly represented by lipoproteins of different origin. In bull semen extenders hen egg yolk is traditionally used (Stradaioli *et al*., 2007). However, due to its animal origin and HDL content, egg yolk could represent sanitary risk, and has a potentially negative impact on sperm PM structures and sanitary risk (Bousseau *et al*., 1998; Amirat *et al*., 2005). Moreover, egg yolk particles may complicate the further analysis of sperm (Ansari *et al*., 2010). Owing to these facts, there has been considerable interest in using extenders without problematic compounds. In bull semen extenders soybean lecithin as phospholipid is the most used alternative to egg yolk, in current practice (Layek et *al*., 2016). Nevertheless, there is no clear consensus concerning its cryoprotective efficacy and suitability in comparison to egg yolk (Gil *et al.*, 2000; van Wagtendonk-de Leeuw *et al.*, 2000; Thun *et al.*, 2002; Aires *et al.*, 2003; Crespilho *et al.*, 2012; Murphy *et al.*, 2017). Another possible substitute for egg yolk is artificially prepared liposomes in the commercially available Optixcell^®^ extender. However, insufficient protective efficacy was proven when compared to the aforementioned extenders (Murphy *et al*., 2017). Further option to overcome problems with egg yolk is using just its cryoprotective constituent – Low Density Lipoprotein (LDL) (Moussa *et al*., 2002). It covers the majority of egg yolk plasma and represents half of phospholipid content (Anton *et al*., 2003) which play the main role in stabilizing PM during cryopreservation (Amirat *et al*., 2005). The precise protective mechanism of soybean lecithin and LDL has been remained unknown (Vidal *et al*., 2013). It was supposed that the mode of action of soybean lecithin is the replacement of lost phospholipids from PM (Zhang *et al*., 2009) or its ability to form a protective layer on PM (Vidal *et al*., 2013). Highly saturated, fast and stable interaction of LDL and BSP (Bovine Sperm Binders) proteins were found (Manjunath *et al*., 2002; Lusignan *et al*., 2011). Prolonged exposure of sperm to these proteins e.g. during different steps of cryopreservation may cause negative changes in plasma membrane which consequently lead to premature capacitation (Faezah *et al*., 2014) or higher sensitivity of sperm to cold shock (Morris *et al*., 2012). Amphipathic molecules of the LDL (Hevonoja *et al*., 2000) have a similar effect on maintaining PM integrity (Bergeron *et al*., 2004). Thereby it may be assumed that these two compounds might work synergistically. Moreover, besides these common characteristics, LDL represents additional extra feature. It contains lipophilic antioxidants as α-tocopherol and ubiquinol (Alvarez-Rodriguez *et al*., 2013) serving as protection against increasing content of Reactive Oxygen Species (ROS) during the cryopreservation process. Similar mechanisms of soybean lecithin and LDL pointed out their presumed synergistic cryoprotective activity. The potential of the common action of LDL and soybean lecithin was determined by our studies, which focused on the prevention cold shock during the equilibration of spermatozoa (Beran *et al.*, 2013; Stadnik *et al.*, 2015; Beran *et al.*, 2016). The preliminary results of this effect during cryopreservation were also positive (Simonik *et al*., 2016), however, this study cover only limited number of animals and sperm parameters.

Therefore, main objective of this study was to confirm the possibility of improvement of bull semen cryopreservation efficacy using LDL as an enhancing factor in soybean lecithin based extenders.

## Material and methods

### LDL extraction

Low-density lipoprotein was prepared with 97% purity according to the methodology of (Moussa *et al.*, 2002) with modification in term of LDL prolonged shelf life. Hen eggs were obtained from the controlled breeding program of BIOPHARM Inc. (Jílové u Prahy, Czech Republic). Compared to the established methodology, slight modifications arose from our previous study (Simonik *et al.*, 2016). Sodium azide (0.1%) was used to preserve the LDL produced and eliminate sanitary risks. Before using the LDL, sodium azide was removed by extensive dialysis against phosphate buffered saline (PBS) (Sigma Aldrich, St. Louis, USA).

### Preparation of the extenders

Soybean lecithin-based extenders, AndroMed^®^ (Minitübe, Tiefenbach Germany) and Bioxcell^®^ (IMV Technologies, L’Aigle, France), were used. Extenders were always prepared fresh just before experiments and strictly according to manufacturer instructions. LDL was then added in selected concentration 6% (v/v) based on our preliminary results (Simonik *et al*., 2016) to each of the aforementioned extenders. Extenders without LDL were used as a control.

### Collection and processing of semen

All experimental work with animals was performed according to EU Directive 2010/63/EU and guidelines of the Czech Legislation (Directive 208/2004 Sb.). Semen was collected from seven bulls (5 ejaculates per bull) ordinarily used for ID production in a standard way and with same frequency of collection (once/week), at the insemination centre (Natural Ltd., Hradištko pod Medníkem, Czech Republic). All sires were of the same age, breed, frequency of collecting and bred under the same management system as related to handling, stabling, feeding system, or feeding ratio composition: hay (10 kg), straw (5 kg), soybean meal (0.5 kg), a mixture of cereals: 1/3 oats, 1/3 wheat, 1/3 barley (3 kg) and the Premin 22 Natural mineral mix (0,1 kg; VVS Verměřovice Ltd., Verměřovice, Czech Republic). Each of 35 ejaculates was submitted to the basic assessment done by trained laboratory technicians from the insemination centre. All ejaculates included in our study match minimal limits of sperm concentration (> 0.7 x 10^9^/ml) and percentage of motile spermatozoa (> 70%). Collected semen was divided into samples in relation to the number of experimental variants and diluted to a final concentration of 120 x 10^6^ spermatozoa/ml. Diluted semen was placed into PVC straws (0.25 ml) and equilibrated for 2 h at 5°C. After this period, straws were cryopreserved using a computerized freezing machine (DigitCool^®^ IMV Technologies, L’Aigle, France) with the standard freezing curve for bovine semen and then immersed directly into liquid nitrogen for storage. Straws were analysed at least one week after the cryopreservation. Before each evaluation, straws were thawed in a water bath for 30 s at 37°C. All analyses were performed after 10 min of sample pre-incubation after thawing at a temperature of 37°C.

### Evaluation of sperm motility

Sperm motility was assessed with the Computer Assisted Sperm Analysis (CASA) module NIS Elements Ar 4.50. (Laboratory Imaging Ltd. Prague, Czech Republic), using a DMK 23UM021 camera (Imaging Source, Germany) with a frame rate of 60 images per second and a stereo microscope (Nikon Eclipse E600, Japan) with a heated plate, magnification x 100. After thawing, semen doses were diluted with Sp-TALP composed according to (Parrish *et al.*, 1988) - 114 mM NaCl, 3.2 mM KCl, 25 mM NaHCO_3_, 0.3 mM NaH_2_PO4.H_2_O, 10 mM HEPES, 10 mM sodium lactate, 2 mM CaCl_2_.2H_2_O, 0.5 mM MgCl_2_.6H_2_O, 6 mg/ml BSA, 1 mM sodium pyruvate, 50 µg/ml gentamycin.

The final concentration of spermatozoa in samples was 20 x 10^6^ spermatozoa/ml (Verstegen *et al*., 2002). Then 10 µl of the sample was evaluated in a Makler^®^ counting chamber (Sefi Medical Instruments, Haifa, Israel) with a 10 µm depth in six different fields per sample. Fields were located strictly out to edges of chamber to avoid the Segre-Silberberg effect. On average, 200 trajectories per field were analysed. Selected kinematic parameters were analysed as follows: curvilinear velocity (VCL, µm/s), velocity of average path (VAP, µm/s), straight line velocity (VSL, µm/s), straightness (STR, %), and amplitude of lateral head displacement (ALH, µm). Percentages of progressively motile sperm (PMOT) were measured by thresholds VAP > 30 µm/s and STR > 70%.

### Flow cytometric assessments

Measurements were made using BD LSR II instrument (Becton Dickinson, San Jose, USA). For FITC (SYBR14) we used 488 nm (20mW) laser line excitation and 525/50 nm emission filter, for PI we used 561 nm (25mW) laser line excitation and 610/20 nm emission filter. For MitoTracker (PE), we used 561 nm (25mW) laser line excitation and 585/15 nm emission filter. Voltages were set for optimum resolution and spectral overlap compensated in BD FACS Diva software using single stain controls and the SW automatic compensation module.

**Figure 3:**
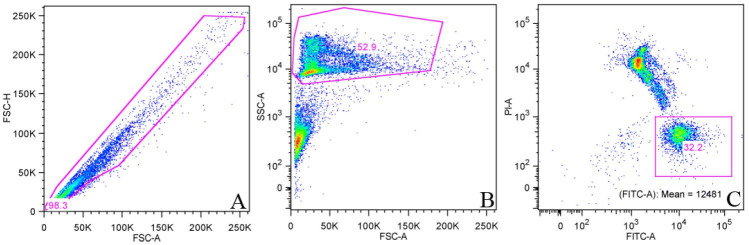
Representative figures of gating strategy and identification of sperm cells during viability analysis using Live/Dead Viability Kit. In the dot plot (A) single cells were gated by plotting FSC-H (linear) vs. FSC-A (linear) to discriminate doublets. Further, single cells were gated for morphology (FSC-A linear vs. SSC-A logarithmic, dot plot B) separation of sperm cells (high SSC signal) from debris (low FSC and SSC signal). Last, we plotted CFDA or SYBR14 (FITC-A) vs. PI (PI-A) in dot plot C) to identify intact (FITC-A positive, PI negative) and dying/dead (FITC-A dim/negative, PI-A positive) sperm cells.

Before each specific sperm parameter evaluation, positive and negative control samples were prepared to proper setting of flow cytometry analysis. Positive controls for SYBR-14 or MitoTracker staining were prepared by standard procedure of Percoll gradient (45/90). Controls for viability staining were prepared according to the methodology of (Petrunkina *et al.*, 2010). Acquisitions were stopped after 30 000 events. Representative dot plots with information about gating, identifying events (sperm cells) are in Figure 3 and Figure 4 in Supplement.

**Figure 4:**
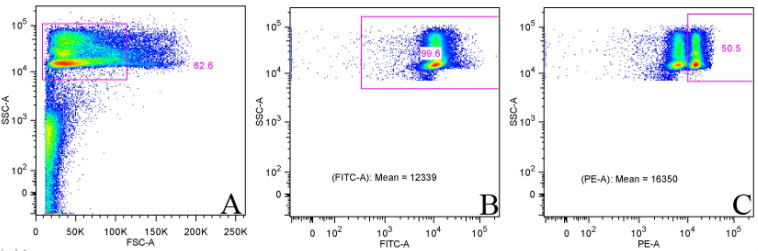
Representative figures of gating strategy and identification of sperm cells during mitochondrial potential analysis using MitoTracker CMXRos. In the dot plot (A), single cells (singlets-not shown) were gated for morphology (FSC-A linear vs. SSC-A logarithmic, plot B) separation of sperm cells (high SSC signal) from debris (low FSC and SSC signal). Then (B) SYBR14 (FITC-A) positive cells were marked as intact, MitoTracker (PE-A) positive cells in plot C were marked as with high mitochondrial membrane potential.

### Sperm viability assessment

For sperm viability analysis, Live/Dead Sperm Viability kit (Life Technologies, Carlsbad, CA) was used. According to the manufacturer manual, SYBR 14 was diluted 50 fold in HEPES buffer saline (10 mM HEPES, 15 mM NaCl, 10% BSA; pH 7,4) and propidium iodide (PI) was used undiluted. After thawing, sperm were diluted with HEPES buffer saline to a concentration 1 x 10^6^ sperm/ml and 5 µl of each fluorochrome were added, resulting in a final concentration of 100 nM of SYBR-14 and 12 µM of PI. The suspension was then incubated in the dark for 10 min at 37°C.

### Assessment of mitochondrial membrane potential

For the evaluation of mitochondrial membrane potential of sperm, MitoTracker Red CMXRos (Invitrogen, California, USA) was used. To distinguish sperm from debris and other particles in extenders, carboxyfluorescein diacetate (CFDA) (Sigma Aldrich, St. Luis, USA) was used. After thawing, sperm were diluted with Sp-TALP to a concentration 1 x 10^6^ spz/ml. Following that, 5 µl of MitoTracker working solution and 1 µl of CFDA were added; final concentration 10 nM and 1 nM, respectively.

### Assessment of acrosomal integrity

For evaluation of acrosomal integrity, *Pissum Sativum* aglutinin conjugated with FITC was used according to methodology (Hu *et al*., 2010). Sperm were diluted with Sp-TALP to concentration 10 x 10^6^ spz/ml and then smeared on microscopic slides. After air-drying, each specimen was washed three times with PBS without Ca^2+^ and Mg^2+^, fixed in methanol (-20°C) for 10 min and again washed three times with PBS without Ca^2+^ and Mg^2+^. Areas for staining were marked with hydrophobic PAP pen (Sigma Aldrich St. Louis, USA) and slides incubated with PSA-FITC (200 mg/ml PBS without Ca^2+^ and Mg^2+^) in a wet chamber for 30 min at 37°C. After washing with PBS without Ca^2+^ and Mg^2+^, Vectashield/DAPI (Vector Laboratories Ltd., Peterborough, UK) was added and the sample was mounted under a coverslip. Evaluation was performed using a epifluorescent microscope at magnification 100x (Nikon Eclipse E600, Tokyo, Japan). Sperm with intact acrosome were characterized with bright homogenous fluorescence in whole acrosome. For each sample, two replicates were evaluated, amounting to 400 spermatozoa counted per sample.

### Statistical analysis

For the evaluation of sperm kinematic parameters from CASA, *k*-means cluster analysis was used to classify motile spermatozoa into subpopulations. Euclidean distances algorithm processed variables STR, VAP, VCL, VSL, ALH with 20 iterations were used to define three clusters (sub-populations) of sperm. According to computed means of selected variables, individual spermatozoon was afterwards assigned to one of three specific sperm subpopulations: fast, medium fast and slow (Tab. 2). To determine differences in the distribution of these subpopulations, the χ2 test was used. The effect of LDL andition in individual groups of extenders (i.e. Andromed – control vs. Andromed + 6% LDL; Bioxcell – control vs. Bioxcell – 6% LDL) was evaluated with Student’s t-test. Data are presented as mean ± SD unless otherwise indicated below. Differences in all cases were considered as statistically significant at P < 0.05. Data are presented as mean +/- SD unless otherwise indicated. Statistical analysis was performed in STATISTICA 12 software (StatSoft, Czech Republic).

**Table 2:** Characterization of different sperm subpopulations determined by cluster analysis of kinematic parameters of motile spermatozoa.

Cluster	N (%)	VCL (μm/s)	VSL (μm/s)	VAP (μm/s)	STR (%)	ALH (μm)
slow spermatozoa	12066 (22.40%)	119.6 ± 30.6	65.6 ± 22.8	72.7 ± 20.4	88.9 ± 14.9	4.6 ± 1.7
medium fast spermatozoa	19988 (37.11%)	208.9 ± 29.2	96.0 ± 22.0	104.9 ± 16.9	91.0 ± 11.7	6.3 ± 1.6
fast spermatozoa	21807 (40.49%)	255.4 ± 28.0	129.2± 22.3	136.3 ± 19.1	94.5 ± 6.7	7.4 ± 2.0

VCL - curvilinear velocity (μm/s), VSL - straight-line velocity (μm/s), VAP - average velocity path (μm/s), STR - straightness (%), ALH - lateral head displacement (μm). Number of observations (n = 35).

## Results

### Effects of LDL addition to soybean based extenders on progressive motility and distribution of sperm subpopulations after thawing

**Figure 1:**
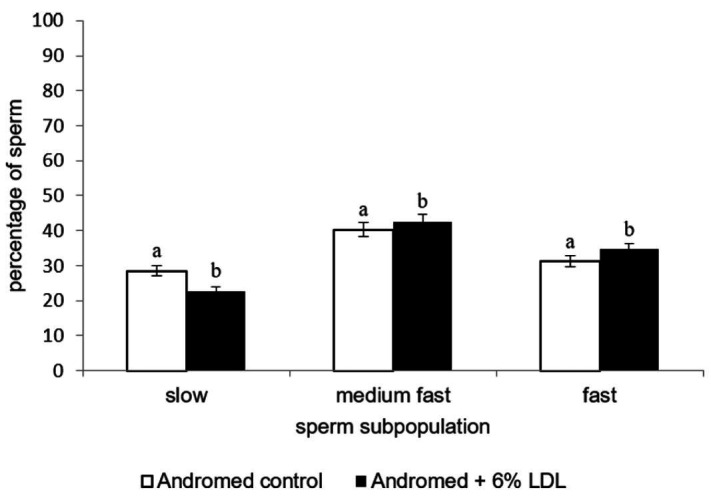
Effect of LDL addition to the Andromed extender on sperm distribution in clusters after thawing. Different superscripts in same cluster represent significant differences (P < 0.05).

**Figure 2:**
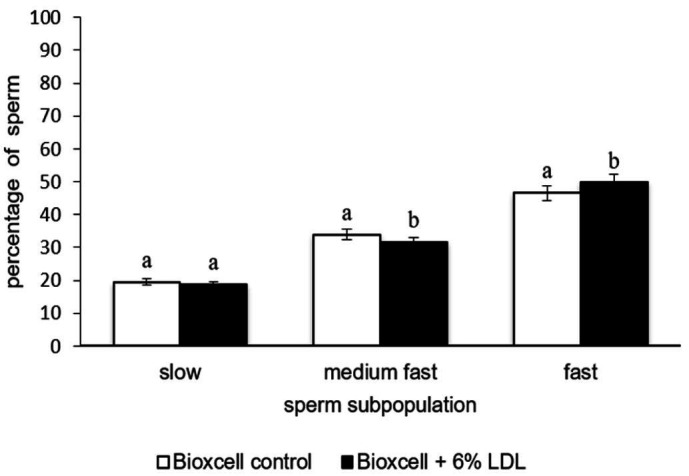
Effect of LDL in Bioxcell extender on sperm distribution in clusters after thawing. Different superscripts in same cluster represent significant differences (P < 0.05).

Sperm motility evaluated as a percentage of progressively motile sperm (PMOT) was not affected by LDL addition in both extenders (P > 0.05) (Tab. 1). Cluster analysis of sperm kinematic parameters (total number of 53,871 spermatozoa) revealed the effect of LDL addition. Based on cluster analysis, the following sperm subpopulations were defined as: slow, medium fast and fast. The summary of number of spermatozoa for each subpopulation and kinematic parameters defining them are given in Table 2. The addition of 6% to AndroMed^®^ and Bioxcell^®^ extenders, increased the percentage of fast spermatozoa subpopulation (AndroMed^®^ control: 31.2%, AndroMed^®^ + 6% LDL 34.6%, Fig. 1, P < 0.05; Bioxcell^®^ control: 46.6, Bioxcell^®^ + 6% LDL: 49.8, Fig. 2, P < 0.05). A significantly reduced percentage of slow sperm was observed only in the case of LDL addition to Andromed (AndroMed^®^ control: 28.5%, AndroMed^®^ + 6% LDL: 22.8, Fig. 1, P < 0.05). Medium-fast sperm subpopulation was affected by LDL addition in both extenders (AndroMed^®^ control: 40.3%, AndroMed^®^ + 6% LDL 42.6, Fig. 1, P < 0.05).

**Table 1:** Effect of LDL addition to soybean lecithin-based extenders on sperm qualitative parameters after thawing. Data are expressed as Mean ± SD.

Experimental group	PMOT (%)	Viability (%)	Acrosome integrity (%)	Mitochondrial membrane potential (%)
Andromed - control	47.7 ± 11.8	32.1 ± 8.4	57.4 ± 13.1	28.9 ± 8.5^a^
Andromed + 6% LDL	48.1 ± 11.7	31.5 ± 7.2	59.2 ± 13.5	32.1 ± 8.9^b^
Bioxcell - control	49.1 ± 9.3	27.3 ± 7.6	56.9 ± 12.1^a^	30.5 ± 10.2
Bioxcell + 6% LDL	50.1 ± 10.0	28.5 ± 8.6	63.3 ± 9.6^b^	31.6 ± 9.1

PMOT – progressively motile; Numbers with different superscripts within a column significantly differ (P < 0.05). Number of observations (n = 35) for each group.

### Effects of LDL addition to soybean based extenders on sperm viability, acrosomal and mitochondrial membrane potential

All following results are presented in Table 1. The effect of LDL addition to the extender AndroMed^®^ nor Bioxcell^®^ on the incidence of spermatozoa with intact PM was not demonstrated (P > 0.05). The percentage of sperm with intact acrosome was improved when LDL was added to Bioxcell^®^ extender (P < 0.05) while in the case of AndroMed^®^ with 6% LDL differences were insignificant (Tab. 1). Mitochondrial membrane potential of cryopreserved sperm was affected by the LDL (P < 0.05). The addition of six percent LDL to AndroMed^®^ extender improved mitochondrial intactness after thawing (Tab. 1, P < 0.05), while in Bioxcell^®^ extender, the differences were insignificant (Tab. 1).

## Discussion

A long period elapsed from the first real experimental evidence that sperm could be cryopreserved (Polge *et al*., 1952). However, sperm quality after thawing is currently considered unsatisfactory, despite the efforts of many research groups to improve it (Srivastava *et al*., 2013). In light of the facts that i) there are inconsistent data on the cryoprotective efficacy of soybean lecithin (SL) based extenders as the most accepted alternative for egg yolk (EY) (Muino *et al*., 2008), ii) LDL is cryoprotective compound of egg yolk (Moussa *et al*., 2002) and iii) main method to improve results of spermatozoa cryopreservation is modification of semen extender composition (Holt, 2000), study is aimed to assess the effect of LDL on SL cryoprotective properties. Our results showed that adding LDL to soybean lecithin-based extenders considerably ameliorated the functional parameters of spermatozoa after thawing. However, the effect of LDL was shown in extender dependent. Motility of spermatozoa was positively influenced in both case of extenders. Acrosome intactness was increased in both groups of extenders, however significantly only in case of LDL to Bioxcell. Regarding effect of LDL on mitochondrial membrane potential, positive significant impact on this parameter was observed in after LDL addition to Andromed.

As has been supposed on the basis of first evidence (Pace *et al*., 1974) and further confirmation in more recent study (Moussa *et al*., 2002) LDL is a functional cryoprotective compound of egg yolk. It was found that LDL positively affects different bull sperm compartments and functions post-thawed e.g. (Amirat *et al*., 2004; Hu *et al*., 2010; Hu *et al*., 2011). All previous studies by other authors dealt only with the possibility of egg yolk substitution by LDL. Attempts to examine the effect of LDL addition to animal protein-free SL extenders when there is commercially available liposome based Optixcell^®^ have been strongly supported by a very current study done by (Murphy *et al.*, 2017). The authors compared the protective properties of Optixcell^®^ with different types of semen extenders. The study found that Optixcell^®^ was not as sufficient as SL and EY extender in protection of sperm during short term storage when progressive motility was evaluated. Moreover, this predominantly field study revealed that the calving rate was highest when EY extender was used for semen preservation. This supports doubts concerning SL and EY extenders effectiveness (Gil *et al.*, 2000; van Wagtendonk-de Leeuw *et al.*, 2000; Thun *et al.*, 2002; Aires *et al.*, 2003; Crespilho *et al.*, 2012; Murphy *et al.*, 2017). The fact that artificial liposome-based extender did not exhibit such protection during semen chilling showed that other methods to improve the cryoprotective properties of bull semen extenders could be found. Our previous studies (Beran *et al.*, 2013; Stadnik *et al.*, 2015; Beran *et al.*, 2016) pointed to the positive effect of LDL addition to SL based extenders already before cryopreservation. Our preliminary results (Simonik *et al*., 2016) indicated the effect of LDL on the sperm motility after cryopreservation. In the present study, a larger variety of functional parameters were assessed in a higher number of replicates. Thereby, more precise insights were provided on the effect of the LDL and its presumable impact on cryoprotective efficacy of SL extenders on sperm quality. Our results showed that in either case of AndroMed^®^ or Bioxcell^®^ extender, LDL addition had a significantly beneficial effect on the percentage of sperm in subpopulations fast, progressive and slow (Fig.1 and Fig. 2). LDL was shown to be more suitable for preserving sperm motility post-thawed in Bioxcell^®^ since, after its addition the samples exhibited the highest percentage of spermatozoa in fast subpopulation. Moreover, as has been confirmed by (Ferraz *et al.*, 2014) the percentage of spermatozoa in this subpopulation correlates with fertilizing ability of sample. Discrepancies in LDL influence on the proportion of medium fast sperm cells across extenders could be caused by possible differences in the content of soybean lecithin in extenders. Nevertheless, this subpopulation represents sperm in a “transient” state. Concerning the results of percentages of progressively motile spermatozoa (PMOT), the effect of LDL was not demonstrated in either extender.This clearly shows the general character of this parameter, however it also demonstrates generally suitable quality of all specimens. Indeed, cluster analysis is widely accepted by the researcher community as a method reflecting the heterogeneity of sperm population in samples (Martinez-Pastor *et al*., 2011); thereby it pointed out more precisely the response of cells to modified conditions during cryopreservation (Holt *et al*., 2004; Muino *et al.*, 2008; Sichtar *et al.*, 2017) and was able to unveil the more subtle effects of LDL on sperm motility. During low temperatures within cryopreservation, LDL is suggested as playing one of the key roles in egg yolk gelation (Wakamatu *et al*., 1982) during which the structure of LDL is disrupted and thus phospholipids, cholesterol and other constituents of LDL (except apoproteins) may be released to the media surrounding sperm. Consequently, phospholipids and cholesterol could interact with PM and form a protective layer (Martinet *et al*., 2003; Amirat *et al*., 2005). Moreover, cholesterol present in the LDL structure could interact deeper in PM structure and finely tune it in the sense of phospholipids: cholesterol ratios that make this structure more resistant to harsh conditions during cryopreservation (Bergeron *et al*., 2004). This fact has been considered by (Oldenhof *et al.*, 2015) as one of the main cryoprotective effects of egg yolk. Higher concentrations of cholesterol in PM can restrict conformational changes of proteins (Sultan *et al*., 2010) and thus keep their biological activity to processes essential for successful fertilization e.g. capacitation (Travis *et al*., 2002) or the sperm-egg interaction (Jankovicova *et al*., 2016). Our results showed no significant positive effect of LDL on sperm PM in either extender (Tab. 1). This is contrary to LDL effect before cryopreservation on PM assessed by our previous study (Beran *et al.*, 2016) and other studies e. g. (Hu *et al*., 2011), which revealed its significant effect on PM but also after thawing. Above all, our results are in compliance with a study (Amirat *et al*., 2005) where the authors provided the closest insight using scanning electron microscopy into the LDL function in PM. It must be born in mind that inconsistencies in results are likely caused by various factors such as methodological approaches of PM integrity analysis. When almost all previous studies dealt with the effect of LDL on bull sperm viability, measuring was accomplished using microscopic techniques. Nevertheless, manifestation of the LDL effect could be specifically hidden in other sperm compartments among others in the acrosome as tightly related to the PM (Perumal *et al*., 2016).

Our results showed the significant positive effect of 6% LDL addition to Bioxcell^®^ on intactness of this compartment (Tab. 1) crucial for successful binding and fertilization of oocytes (Layek *et al*., 2016). Importantly, the same concentration of LDL in the same extender was confirmed as significantly favourable for PM integrity after collection (Beran *et al*., 2016). This is supported by evidence that LDL had a positive indirect influence on maintaining the functional status of PM due to its high binding affinity to BSP proteins (Lusignan *et al*., 2011), thereby preventing destabilization (Srivastava *et al*., 2013). The favourable effects of BSP sequestration on sperm functional parameters were already proven after semen collection (Srivastava *et al*., 2012) and post-thawed as well (Manjunath *et al*., 2002). Our results differ according to the extender used, which could indicate their distinct composition. Moreover, our previous study (Simonik *et al*., 2016) and study of Murphy *et al.* (2017) revealed the distinct effectiveness of LDL across different soybean extenders and protection by themselves alone, respectively. Unfortunately, information about either the precise content of soybean lecithin or other components in extenders is unavailable in commercial extenders. Even more, as Futino *et al.* (2010) stated, excessive concentrations of soybean lecithin are not beneficial for sperm. Due to the fact that LDL also contains lecithin (phosphatydilcholine) (Hevonoja *et al*., 2000) total concentrations of this compound could reach levels inhibiting the positive effects of LDL.

The high content of polyunsaturated fatty acids in the PM is responsible for higher susceptibility to lipid peroxidation by Reactive Oxygen Species (ROS) (Tapia *et al*., 2012). Moreover, as sperm have a limited capacity of antioxidative system (Gharagozloo *et al*., 2011) the positive effect of LDL on these protective characteristics of semen (Hu *et al*., 2011) is significantly important. More specifically, it was determined that LDL increased the activity of catalase, superoxide dismutase, glutathione peroxidase and reduced glutathione (Hu *et al*., 2011). Sperm mitochondria are organelles, which are the most susceptible to the higher levels of ROS usually achieved during cryopreservation (Sieme *et al*., 2008; Ferrusola *et al*., 2009). Currently up to a half of the motile sperm population is irreversibly damaged and lost during cryopreservation (Layek *et al*., 2016). However, these losses could be higher since there is a proportion of sperm affected by sub-lethal damage localized on mitochondria, which extends sperm populations with lower fertilization ability due to energy production loss and a high predisposition to programmable cell death (Ferrusola *et al*., 2009). Moreover, the population of dying sperm was determined as a detrimental factor for other intact cells (Roca *et al*., 2016). The results of our study revealed a significant positive effect of LDL on mitochondria integrity, which confirms its presumed synergism with soybean lecithin on antioxidant activity. Our results are in accordance with studies, when LDL was used as a substitution of egg yolk (Hu *et al*., 2011; Perumal *et al*., 2016) and indicated that it could also work in soybean based extenders. It conserved a better mitochondrial function of sperm after thawing, thereby providing better conditions for sperm and leading to a higher probability of fertilization. However, in our study a significant effect on mitochondrial membrane potential was only shown in the case of LDL additions to AndroMed^®^ extender. Inconsistency in effect of LDL addition through extenders might be attributed to the supposed different composition of extenders where final concentration phosphatidylcholine could be over a non-specified limit.

## Conclusion

We suggest that LDL works synergistically and positively interacts with soybean lecithin in selected bull semen extenders. Thereby, LDL preserves functional parameters of bull spermatozoa on higher levels after thawing. However, this depends upon the type of SL extender used. Therefore, future research should be directed to determination the most appropriate combination, mainly by an analysis of the positive effect of specific LDL additions to SL on the fertilizing ability of spermatozoa under field conditions.
